# RORγt Modulates Macrophage Recruitment during a Hydrocarbon Oil-Induced Inflammation

**DOI:** 10.1371/journal.pone.0079497

**Published:** 2013-11-15

**Authors:** Qi Wu, Xin Sun, Ruo Chi, Long Xu, Xue Li, Jing Feng, Huaiyong Chen

**Affiliations:** 1 Tianjin Haihe Hospital, Tianjin Institute of Respiratory Diseases, Tianjin, China; 2 Respiratory Department of Tianjin Medical University General Hospital, Tianjin, China; University of Hong Kong, Hong Kong

## Abstract

Hydrocarbon oils are often utilized as adjuvants in vaccines. In response to naturally occurring hydrocarbon oils, inflammation is initiated and persists with the continuous recruitment of immune cells such as macrophages and neutrophils. However, the mechanism underlying the chronic inflammation in response to hydrocarbon oils is not fully defined. In this study, we revealed an essential role of retinoid-related orphan receptor gamma t (RORγt) in sustaining the recruitment of macrophages following pristane treatment. RORγt absence resulted in the incompetent formation of mesenteric oil granulomas which may associate to a reduction in the migration of macrophages into the mesentery during pristane-induced inflammation. This is at least partially dependent on the expression of the monocyte chemoattractant protein-1 (MCP-1) in the mesentery and the decrease in the macrophage reservoir in the spleen. However, the absence of RORγt had no impact on the recruitment of neutrophils to the mesentery after pristane treatment. Our data uncovered an important role of RORγt in the recruitment of macrophages during hydrocarbon oil-induced chronic inflammation.

## Introduction

Exposure to naturally occurring hydrocarbon oils is associated with the development of a variety of pathologies in animal models and humans [1], [2], [3], [4]. Due to their ability to promote and sustain inflammation, hydrocarbon oils are often utilized as adjuvants in the development of vaccines. Pristane (2,6,10,14-tetramethylpentadecane) represents one of the most studied hydrocarbon oils. When injected intraperitoneally, pristane is sequestered by inflammatory leukocytes to form cell-oil aggregates, which adhere onto the mesentery and result in the formation of mesenteric oil granulomas [5]. Depending on the genetic background, plasmacytomas develop during pristane-induced inflammation [6], [7]. Moreover, pristane was also found to induce lupus-like autoimmune diseases such as glomerulonephritis, arthritis and pulmonary vasculitis in mice [8], [9], [10], [11], [12]. Regardless of the outcomes, chronic inflammation is the common feature among pristane-induced pathologies, which can be characterized by the continuous recruitment of leukocytes including lymphocytes, macrophages and neutrophils [5], [13], [14].

Previous studies from our group and other labs have begun to uncover the mechanisms responsible for the chronic inflammation induced by pristane. We previously demonstrated that B lymphocytes, but not T lymphocytes, are critical for controlling pristane-induced inflammation B lymphocytes promote the sequester of injected pristane into the form of oil granulomas [15]. Although the recruitment of B lymphocytes to the mesentery was intact in mice deficient for the inflammatory cytokine tumor necrosis factor alpha (TNFα), in response to pristane, the formation of oil granulomas was still defective [15]. This finding was accompanied by a reduced recruitment of macrophages and neutrophils in the mesentery, which suggests that macrophages and neutrophils also play an important role in controlling pristane-induced inflammation. Cytokines and chemokines are known to modulate the migration of macrophages and neutrophils during inflammation. For example, type 1 interferon promotes the migration of macrophages into the peritoneum by activating the expression of chemokines, including monocyte chemoattractant protein 1 (MCP-1), after pristane treatment [13]. Interleukin 1 alpha (IL1α) and the IL-1 receptor promote the migration of neutrophils to the peritoneal cavity in a CXC chemokine receptor-2-dependent manner [16]. The retinoid-related orphan receptor gamma t (RORγt), which is important for the development of Th17 cells and the organogenesis of lymphoid organs [17], [18], [19], was recently reported to be able to induce steroid-insensitive neutrophilic airway inflammation by enhancing Th17 cell differentiation and IL17 cytokine production [20]. IL17 cytokine production is dependent on Toll-like receptor 4 in pristane-induced experimental lupus [21]. IL17 is able to recruit macrophages via the expression of MCP-1 in rheumatoid arthritis synovial fibroblasts and macrophages [22]. Nonetheless, it remains unknown whether RORγt plays a role in hydrocarbon oil-induced chronic inflammation.

The present study examined the possible roles of RORγt in pristane-induced inflammation. Our data suggest that RORγt modulates the recruitment of macrophages to the inflammation site by altering both the reservoir of macrophages in the spleen and the expression level of MCP-1 in the mesentery. However, RORγt exhibited little effect on the migration of neutrophils. This preliminary study implicates a novel role of RORγt in the organization of oil granulomas during inflammation.

## Materials and Methods

### Ethics Statement

Mice between the ages of 2–4 months were used in strict accordance with the protocol approved by the Nankai University Animal Care and Use Committee.

### Mice and Treatments

Mice with green fluorescent protein reporter complementary DNA knocked-in at the initiation site of RORγt translation on the C57BL/6 background (RORγt^−/−^) and C57BL/6 (WT) mice were purchased from Jackson Laboratory (Bar Harbor, ME, USA). The mice were housed at the Nankai University Animal Care Facility under specific pathogen-free conditions with sterile bedding, water, and food. At the age of eight to ten weeks, the mice received a single intraperitoneal (i.p.) injection of pristane (300 µl; 8.3×10^−4^ mol) (≥95% pure, Sigma-Aldrich, St Louis, MO, USA). Age-matched, untreated mice were used as controls. Mice were used in strict accordance with the protocol approved by the Nankai University Animal Care and Use Committee.

### Tissue Isolation and Cell Collection

As previously described [14], whole mesenteric tissue (WMT) from the distal duodenum through the terminal ileum was removed intact from naϊve and pristane-treated mice via dissection. The WMT was spread out in ice-cold PBS and photographed with a Canon camera (EOS 20D) equipped with a macro-lens (EF-S 60 mm). Single cell suspensions were prepared via digestion of the recovered tissue in RPMI 1640 medium that contained 0.5 mg/ml type I collagenase (Sigma-Aldrich), 0.5 mg/ml type IV collagenase (Sigma-Aldrich), 0.2 mg/ml deoxyribonuclease I (DNase I, Sigma-Aldrich), and 25 mM HEPES buffer in Erlenmeyer flasks with constant stirring. The tissue digestion was carried out at room temperature for 1 h; the resulting cell suspension was removed and filtered through fine nylon mesh (Denville Scientific Inc., Metuchen, NJ, USA). Cells were then washed and resuspended in buffer (HBSS +5% FBS) for flow cytometric analysis or chemokine expression analysis by quantitative PCR. In indicated experiments, 5-mm biopsy punches from the granulomas in the center of the mesenteric tissue (MG) of the pristane-treated mice or the corresponding area in the naïve mice were excised. The individual nodules developed along the border of the mesentery and gut (SG) in the pristane-treated mice and the corresponding area in the naïve mice were removed separately by a 2-mm punch. Cells in these tissues were extracted using the same collagenase/DNase I solution that was used to extract cells from the WMT. Resident cells in the peritoneal cavity (PC) were collected by lavage with 10 ml of ice-cold RPMI supplemented with 5% FBS. After centrifugation, cell pellets were harvested for flow cytometric analysis. Cell samples were also obtained from the spleen and right femur for flow cytometric analyses as described previously [14].

### Analysis of Cell Types by Flow Cytometry

Cells harvested from the WMT, spleen, bone marrow (BM) or PC were incubated on ice with ammonium chloride buffer for 1 min to lyse red blood cells before labeling. Approximately 10^6^ cells were incubated on ice with FcR (CD16/32) blocking antibody for 20 min, washed, and then labeled (30 min) on ice with antibodies specific for B220 (PE-Cy7), TCRβ (APC), CD11c (PE), Gr-1 (FITC), and CD11b (APC-Cy7). Propidium iodide (Sigma-Aldrich) was included to discriminate dead cells. Labeled cells were analyzed in a FACSVantage with DIVA option.

### Quantitative PCR

Total RNA was extracted from approximately 10^6^ cells using TRIzol reagent (Invitrogen, Carlsbad, CA, USA). Messenger RNA was reverse transcribed (Superscript III; Invitrogen) with oligo (dT) primer for 1 h at 50°C. Quantitative PCR was performed in an iCycler thermal cycler (Bio-Rad Laboratories, Hercules, CA, USA) with SYBR Green PCR core reagents (Applied Biosystems, Foster City, CA, USA) and primers for specific genes. The following primers were used: β-actin, forward, 5′-AGCCATGTACGTAGCCATCC-3′, and reverse, 5′-CTCTCAGCTGTGGTGGTGAA-3′; MCP-1, forward, 5′-CTTCTGGG CCTGCTGTTCA-3′, and reverse, 5′-CCAGCCTACTCATTGGGATCA-3′; and CXCL2, forward, 5′-AGTGAACTGCGCTGTCAATG-3′, and reverse, 5′-AGGCACATCAGGTAGGATCC-3′. The amplification conditions were as follows: an initial cycle of heating at 94°C for 10 min, followed by 40 cycles of amplification at 94°C for 15 s and 60°C for 45 s. The relative levels of mRNA for specific target genes were calculated by the comparative Ct (threshold cycle) method, which was normalized to the β-actin in the same sample, following the manufacturer’s instructions (Applied Biosystems).

### Statistics

Differences between paired groups were analyzed using two-tailed Student’s t-test; P values ≤0.05 were considered significant.

## Results

### Effect of RORγt on the Formation of Pristane-induced Oil Granulomas

**Figure 1:**
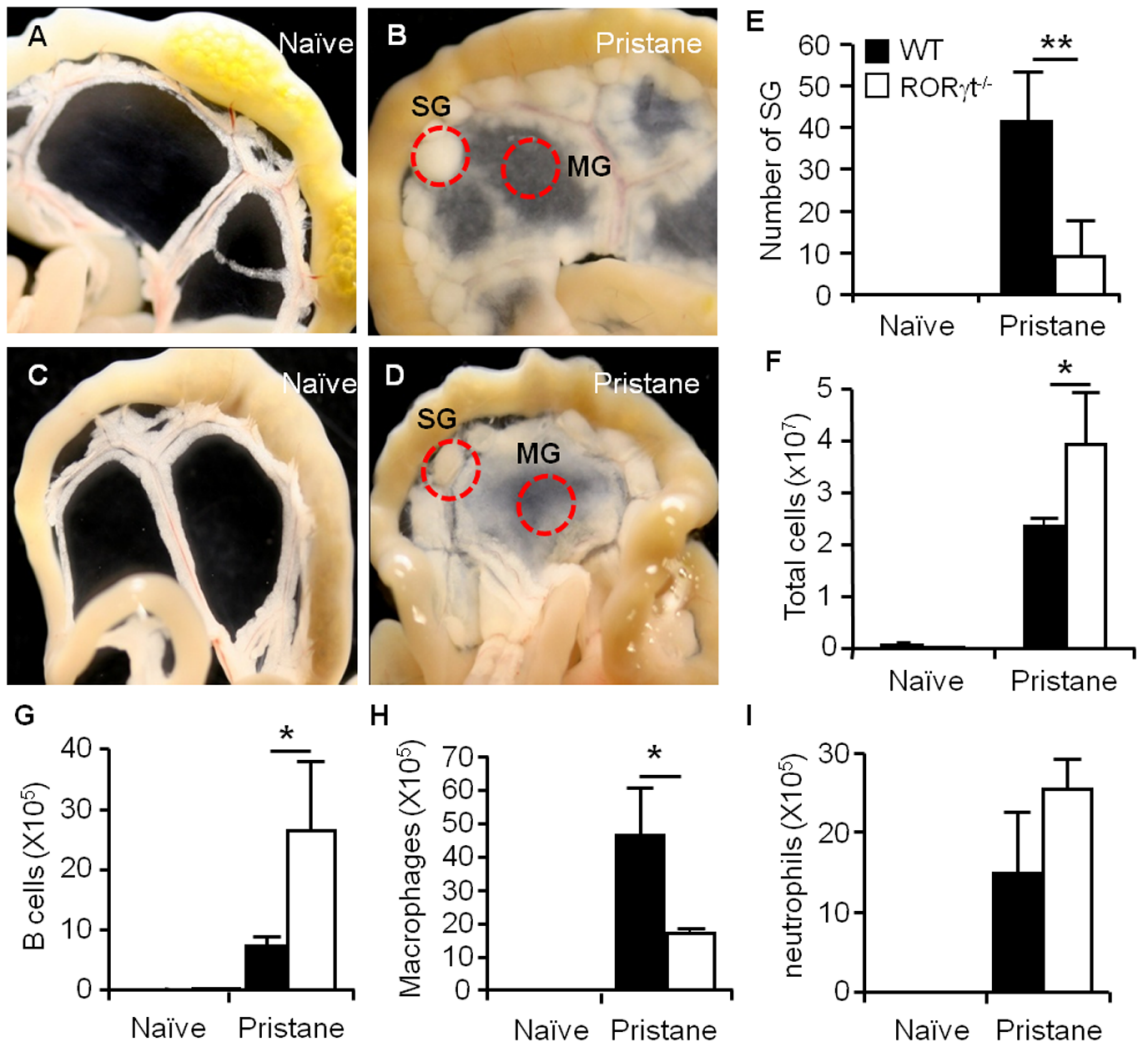
RORγt controls pristane-induced formation of oil granulomas on the mesentery. (A–B) The mesenteric tissues associated with the gut were collected from naïve mice or pristane-treated C57BL/6 mice (3 weeks) and photographed. SG, serosal granulomas; MG, mesenteric granulomas. (C–D) Images shown represent the mesenteric tissues associated with the gut in either naïve or pristane-treated (3 weeks) RORγt^−/−^ mice. (E) SG was quantitated in wild type (WT) controls and RORγt^−/−^ mice after pristane treatment. (F), Analysis of cellularity of whole mesenteric tissue in WT and RORγt^−/−^ mice. (G-I), The number of B cells (B220^+^), macrophages (CD11b^+^Gr1^low^) or neutrophils (CD11b^+^Gr1^hi^) in whole mesenteric tissue was summarized. Data show the average ± SD of three mice, representing three independent experiments. *p<0.05, **p<0.01, compared with the WT group.

The chronic inflammation induced by intraperitoneal injection of the hydrocarbon oil pristane can be characterized by the development of mesenteric oil granulomas. In untreated mice, the mesentery was found to be thin and clear (Figure 1A). Consistent with data reported previously [5], [14], the mesentery became milky three weeks after pristane treatment and exhibited the formation of two classes of oil granulomas: granulomas about the center of the mesenteric tissue and away from peripheral fat and blood vessels (MG), and individual SG that developed along the border of the mesentery and intestine (Figure 1B). Using flow cytometry (Figure S1 and [14]), we observed that the cells in oil granulomas included lymphocytes, macrophages and neutrophils. Inflammation began to resolve by week 11 after one dose of pristane, which was evidenced by the disappearance of the SGs and the resolving of the MG [14]. Therefore, all subsequent experiments were performed at 3 weeks after pristane injection. The development of oil granulomas resulted from the continuous adherence of aggregates of oil and peritoneal-infiltrated leukocytes onto the mesentery. We demonstrated that pristane induced a dramatic expansion of myeloid cells, which included macrophages (CD11b^+^Gr1^low^) and neutrophils (CD11b^+^Gr1^hi^), in the peritoneal cavity (Figure S2). They comprised a large portion of the peritoneal cell infiltrate (Of total, CD11b^+^Gr1^low^: ∼32%; CD11b^+^Gr1^hi^: ∼38%) (Figure S2).

RORγt is known to play a crucial role in the development of some secondary lymphoid tissues and the progression of ovalbumin-induced neutrophilic inflammation [18], [19], [20]. Here, we asked whether RORγt is involved in the formation of pristane-induced oil granulomas. To answer this, the RORγt^−/−^ mice and the wild-type (WT) C57BL/6 controls were treated with pristane. After pristane treatment, the RORγt^−/−^ mice showed a deposition of oil-cell aggregates on the mesentery during pristane-induced inflammation (Figure 1D). However, the number of the SG in the RORγt^−/−^ mice was approximately one fourth of the number observed in the WT controls (Figure 1E), which suggests that RORγt is important for the development of SG. Although there was no difference in the accumulation of inflammatory cells in the peritoneal cavity of RORγt^−/−^ mice versus WT controls (data not shown), the absence of RORγt promoted the accumulation of leukocytes in the mesentery (Figure 1F). This accumulation was largely due to the increase in the number of B220^+^ cells (Figure 1G), which was paralleled by a significant reduction of macrophages and no change in neutrophils in the absence of the RORγt signal (Figure 1H–I).

### RORγt Modulates the Leukocyte Recruitment during Pristane-induced Inflammation

**Figure 2:**
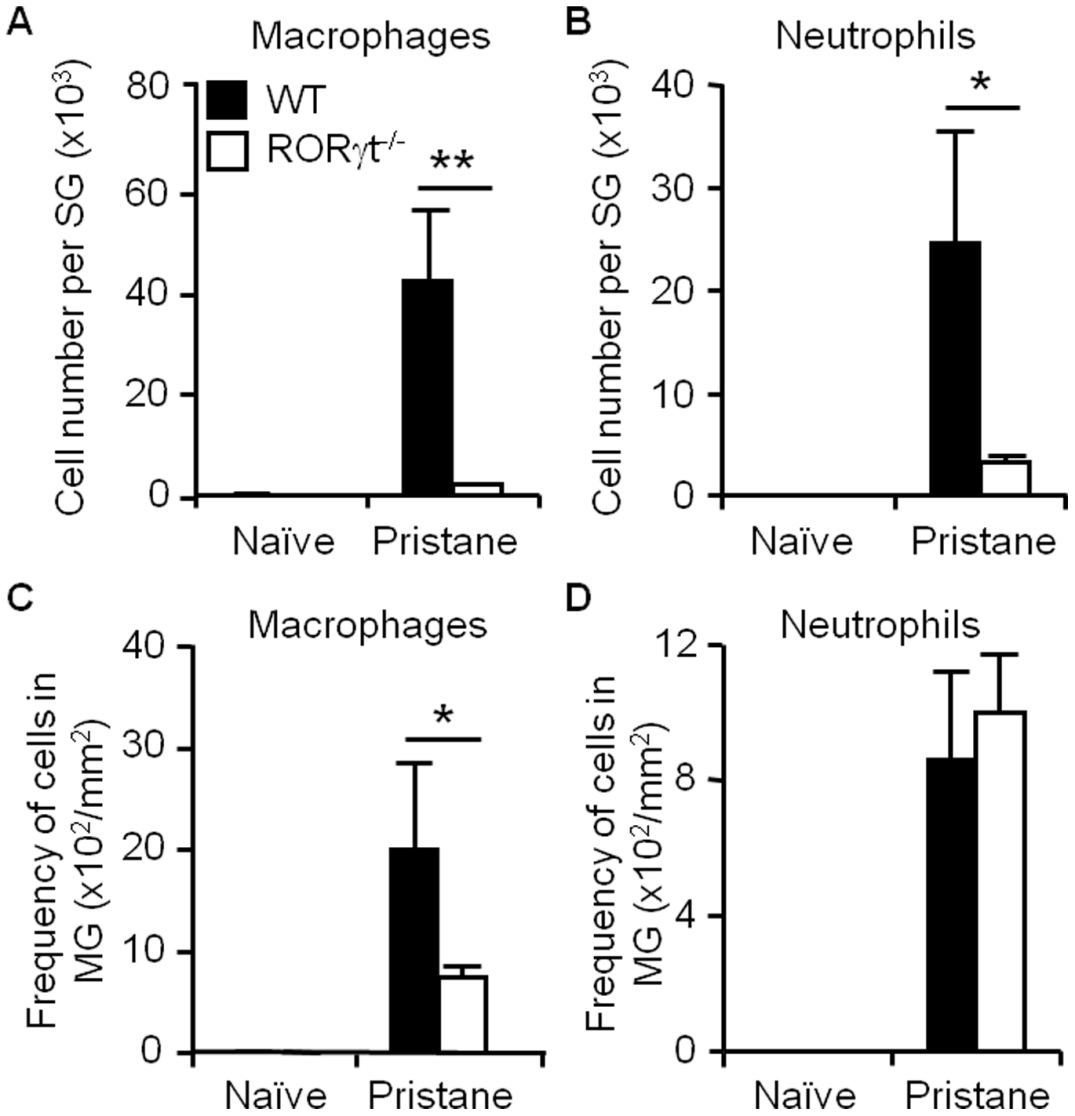
RORγt deficiency blocks macrophage recruitment to oil granulomas. (A–B), Recruitment of macrophages and neutrophils to SG; data are shown as the absolute numbers of each cell type per individual SG. (C–D), Recruitment of macrophages and neutrophils to MG; data are shown as the absolute numbers of each cell type per mm^2^ of MG. *p<0.05, **p<0.01, compared with the WT group.

Inflammatory leukocytes assist in walling off pristane, thereby limiting pristane-induced pathologies. We investigated the effects of RORγt on leukocyte recruitment to the SG or MG. For the SG, the recruitment of macrophages and neutrophils was almost blocked in the absence of RORγt (Figure 2A–B). Similar to the SG, the recruitment of macrophages to the MG was inhibited in the absence of RORγt (Figure 2C). However, RORγt had no impact on neutrophil recruitment to the MG (Figure 2D). Collectively, these data suggest that RORγt modulates macrophage recruitment in the pristane-induced formation of mesenteric oil granulomas.

### RORγt Alters the Expression of MCP-1 in the Mesentery

**Figure 3:**
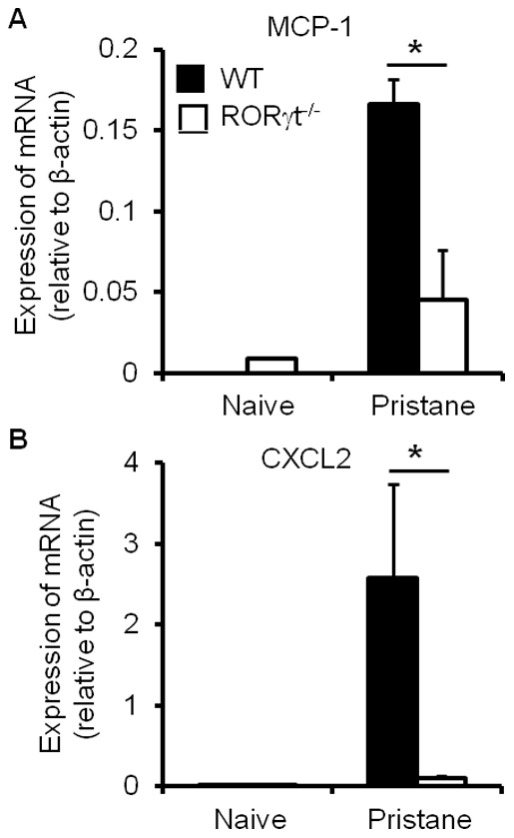
RORγt deficiency alters the expression of chemokines by mesenteric cells during pristane-induced inflammation. Levels of mRNA of indicated chemokines in mesenteric cells from naïve or pristane-injected WT and RORγt^−/−^ mice were measured by quantitative PCR. Data show the average ± SD of three samples, representing three independent experiments. *p<0.05, compared with the control group.

It has been demonstrated that chemokines are responsible for myeloid cell migration. We reasoned that chemokine production may be altered in the absence of RORγt during pristane-induced inflammation. We observed a significant reduction in the mRNA expression level of monocyte chemoattractant protein-1 (MCP-1) by mesenteric cells in RORγt^−/−^ mice compared with the WT controls (Figure 3A). This finding may support the observation that RORγt promotes macrophage recruitment to the mesentery after pristane treatment. However, the expression of CXCL2, which mediates the migration of neutrophils, followed a similar pattern in the absence of RORγt (Figure 3B), although RORγt had no impact on the recruitment of neutrophils during pristane-induced inflammation (Figure 1I). This discrepancy suggests that the recruitment of neutrophils, or even other inflammatory leukocytes, may be also dependent on factors other than chemokine expression.

### Effect of RORγt on the Reservoir of Leukocytes in the Spleen during Pristane-induced Inflammation

**Figure 4:**
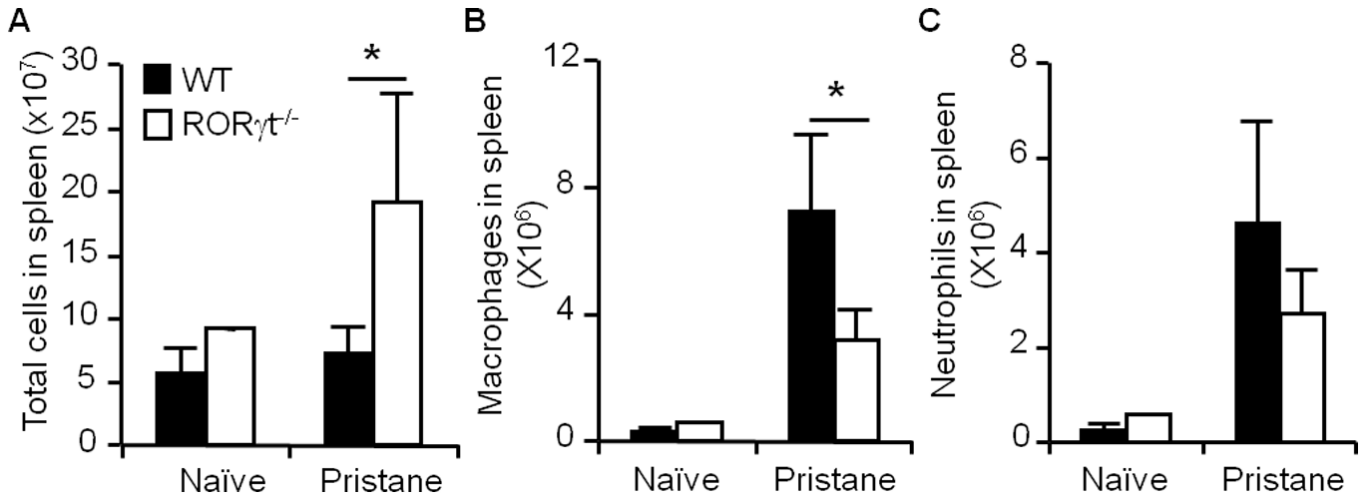
RORγt deficiency reduces splenic macrophage expansion during pristane-induced inflammation. Total cells (A), macrophages (B) and neutrophils (C) in the spleens of naïve or pristane-treated WT or RORγt^−/−^ mice were numerated. Data show the average ± SD of three samples, representing three independent experiments. *p<0.05, compared with the control group.

The spleen is a site known for the storage and rapid deployment of leukocytes during inflammation [23]. Therefore, we asked whether the effects of RORγt deficiency on myeloid cell recruitment to the mesentery after pristane treatment are also determined by the size of the reservoir of myeloid cells in the spleen. In concordance with previous reports [24], we found an increase in splenic cellularity in the absence of RORγt at steady-state (Figure 4A). However, RORγt deficiency had no impact on the number of macrophages or neutrophils (Figure 4B–C) at steady-state. Three weeks after pristane treatment, a further increase was observed in the total cellularity in the spleens of RORγt^−/−^ mice (Figure 4A). However, macrophages was expanded to a less extend in the spleens of RORγt^−/−^ mice compared with the WT controls (Figure 4B), which indicates that fewer splenic macrophages were available to mobilize towards the inflamed mesenteric tissue. Neutrophils, unlike macrophages, exhibited comparable expansion in the spleen when responding to pristane treatment in the WT controls and RORγt^−/−^ mice (Figure 4C). These data correlated with the accumulation of macrophages and neutrophils in the mesenteric tissue during pristane-induced inflammation (Figure 1H–I).

## Discussion

Hydrocarbon oils are commonly incorporated as adjuvants during the development of vaccines to augment the response to immunization [25], [26]. However, exposure to hydrocarbon oils is also associated with the development of chronic inflammation and a number of pathologies including granulomas, plasmacytomas and autoimmune manifestations [1], [2], [3], [4].

The granulomas act as a physical barrier that walls off substances. The disruption of granuloma structures can lead to the spread of the isolated substances, which results in further reactivation of diseases associated with the inducing stimuli [5], [27]. Thus, it is crucial to understand the mechanisms that underlie hydrocarbon oil-induced chronic inflammation and granuloma formation.

Among the most potent hydrocarbon oils that induce chronic inflammation is the medium-length alkane pristane. After intraperitoneal injection of pristane, a variety of leukocytes are recruited into the peritoneal cavity and later migrate to the mesentery [14]. Those inflammatory leukocytes include macrophages, neutrophils, dendritic cells, B lymphocytes and T lymphocytes [15], [28]. Small volumes of pristane are phagocytosed by macrophages, while larger volumes of pristane become surrounded by neutrophils and other inflammatory leukocytes to form oil-cell aggregates that adhere to peritoneal surfaces, which result in the formation of oil granulomas [5]. Therefore, macrophages and neutrophils play a critical role in controlling the development of inflammation because of their ability to entrap pristane. Defects in the recruitment of macrophages and neutrophils will result in inefficient development of oil granulomas and thereby prolong inflammation in the peritoneal cavity. This phenomenon is supported by observations that when responding to pristane, fewer macrophages and neutrophils are recruited to the mesentery in B cell deficient mice or TNFα knockout mice, which results in the defective formation of oil granulomas [15]. Depending on the genetic background of mice, oil granulomas may represent a form of ectopic lymphoid tissue [27].

RORγt is a transcription factor that regulates the development of lymph nodes and Peyer’s patches, the development of Th17 cells, and the progression of airway neutrophilic inflammation [17], [20], [29]. In this study, we reported that RORγt promotes the formation of oil granulomas during pristane-induced inflammation by accelerating the recruitment of macrophages to the mesentery. However, RORγt deficiency has no impact on the recruitment of neutrophils to the MG. The recruitment of macrophages to the inflammatory site is dependent on both the expression of corresponding chemokines and the reservoir of macrophages in lymphoid organs such as the spleen. This finding was supported by our observation that RORγt^−/−^ mice displayed less expansion in splenic macrophages and less MCP-1 expression in the mesentery compared with the wild type controls after pristane treatment. Macrophages migrate into the peritoneal cavity via circulation during inflammation. Therefore, we predicted that there are fewer circulating macrophages in RORγt^−/−^ mice compared with the wild type controls during pristane-induced inflammation; this finding should be examined further.

RORγt induces the expression of Th17 cytokines including IL17, IL21 and IL22, which promotes tissue inflammation by the induction of other proinflammatory mediators and by the recruitment of leukocytes to the sites of inflammation. For example, IL17 is able to recruit macrophages via the expression of MCP-1 in rheumatoid arthritis synovial fibroblasts and macrophages [22]. IL17 induction of MCP-1 was mediated by phosphoinositide 3-kinase (PI3K) extracellular signal-regulated kinase (ERK) pathways in macrophages [22]. Based on the published data and our current findings, we hypothesized that RORγt modulates pristane-induced inflammation through IL17 production, which recruits macrophages to the inflamed mesentery via MCP-1 expression.

T cells were shown to express RORγt [30], [31], but mice that lacked T cells were still able to develop oil granulomas competently [15]. This finding suggests that the RORγt signal on T cells is dispensable for the development of mesenteric oil granulomas during pristane-induced inflammation. In addition to T cells, macrophages were also shown to express RORγt [32]. The present study was unable to uncover the mechanisms by which pristane activates RORγt in macrophages and how the RORγt signal increases the number of macrophages in the spleen. We also could not determine if the RORγt signal is what attributes to the formation of oil granulomas instead of local or circulating macrophages. These questions should be addressed in the future.

## Supporting Information

Figure S1 **Identification of cell infiltrate in mesentery after pristane treatment by flow cytometry.** Single cell suspensions prepared from the spleens of pristane injected C57BL/6J mice (300 µl pristane, 3 weeks) were stained and analyzed by flow cytometry. (A–B) Live cells were positively selected for analysis of CD11c expression, which results in identification of CD11c^+^ DCs and CD11c^−^ fraction. (C) Analyzing the expression of B220 of CD11c^−^ cells revealed B220^+^ fraction (conventional B cells). (D) The CD11c^−^B220^−^TCRβ^−^ fraction was furthered fractionated by the expression of CD11b and Gr-1 into CD11b^+^Gr-1^low^ (macrophages) and CD11b^+^Gr-1^hi^ (neutrophils).
(TIF)

Figure S2 **Influx of inflammatory leucocytes into the peritoneal cavity following pristane injection.** Peritoneal cells were harvested from naïve or pristane injected C57BL/6J mice at 3 weeks. Numbers of total cells (A), B cells (B), macrophages (C) and neutrophils (D) in peritoneal cavity were analyzed by flow cytometry.
(TIF)
